# Evaluation of severe adverse cutaneous drug reactions in patients admitted to tertiary care center: A cross‐sectional study

**DOI:** 10.1002/hsr2.1969

**Published:** 2024-03-13

**Authors:** Mohammad Amin Moshayedi, Ali Asilian, Fatemeh Mokhtari

**Affiliations:** ^1^ School of Medicine Isfahan University of Medical Sciences Isfahan Iran; ^2^ Department of Dermatology, School of Medicine, Skin Diseases and Leishmaniasis Research Center Isfahan University of Medical Sciences Isfahan Iran

**Keywords:** complication, dermatology, drug hypersensitivity syndrome, Stevens‐Johnson syndrome

## Abstract

**Background and Aims:**

Adverse cutaneous drug reactions (ACDRs) are common and potentially life‐threatening, while also hindering patient compliance to medications. Given the regional differences in patterns and prevalence of ACDRs, it is important to study the epidemiology, as well as the clinical and outcome patterns of patients with ACDRs in Iran.

**Methods:**

This cross‐sectional study on ACDRs was conducted among hospitalized patients in a referral university hospital in the city of Isfahan, Iran. The patients' demographics, clinical information, and outcomes, including age, gender, past medical history, medication history, drug reaction with eosinophilia and systemic symptoms (DRESS) diagnosis, Steven‐Johnson Syndrome (SJS) diagnosis, toxic epidermal necrosis (TEN) diagnosis, treatment regimen (steroids or intravenous immunoglobulin [IVIG]) and outcome information, including intensive care requirements, severe medical complications, or death, were obtained from medical records.

**Results:**

A total of 195 patients with a mean age of 40 years and consisting of 61% females were included. Carbamazepine, lamotrigine, sodium valproate, and phenytoin are the most commonly reported medications. Rate of complications was 45% with DRESS, SJS, and TEN diagnosed in 26%, 47%, and 19%, respectively. Treatment was carried out with steroids and IVIG in 81% and 19%, respectively. Among patients, 15% required intensive care and 5% died. Diagnosis of TEN, older age, and baseline heart disease were predictors of mortality. Patients with SJS were younger and more likely to be males, and they were more likely to have eye complications. On the other hand, patients with the diagnosis of TEN were more likely to receive IVIG and intensive care, and had a higher mortality rate.

**Conclusion:**

Our study provides insight into the demographics and clinical patterns of Iranian patients with ACDRs. This will help in predicting rates of complications, treatments, and outcomes in patients and therefore make proper management decisions.

## 1 INTRODUCTION

Adverse cutaneous drug reactions (ACDRs) are common and potentially life‐threatening and present in a spectrum, most commonly as mild forms of maculopapular eruptions, fixed drug reaction, angioedema, urticaria, or morbilliform rashes, while occurring as high as 8% of the population and observed in 2%–3% of hospitalized patients.^1^, ^2^, ^3^, ^4^, ^5^, ^6^, ^7^, ^8^ More severe cutaneous forms of drug reaction are often present as Stevens‐Johnson syndrome (SJS) and toxic epidermal necrolysis (TEN) or drug‐reaction with eosinophilia and systemic symptoms (DRESS) with a mortality rate of 5%–50% and 10%–20%, respectively.^9^, ^10^

ACDRs are commonly caused by antiepileptic drugs, antibiotics, and nonsteroidal anti‐inflammatory drugs (NSAIDS).^1^, ^11^, ^12^ ACDRs in their milder form affect patient's compliance with medications and complicate the management of seizure disorders, infectious diseases, or auto‐immune disorders. More severe ACDRs have a high rate of morbidity or mortality.^13^ Therefore, epidemiological studies on clinical patterns, severity, and association with medication profile and background medical issues are crucial in preventing and treating ACDRs. There are reports on the influence of regional epidemiological factors and genetics on the frequency and clinical patterns of ACDRs.^14^, ^15^, ^16^, ^17^ However, given the regional differences in patterns and prevalence of ACDRs, we aimed to conduct a cross‐sectional study on ACDRs in hospitalized patients in a referral university hospital in the city of Isfahan, Iran. We determined the demographic information, background medical problems, drugs associated with adverse reactions clinical patterns of adverse cutaneous in patients hospitalized with ACDRs in our center. This information will help in understanding the clinical patterns of patients with ACDRs in Iran and help in predicting rates of complications, treatments, and outcomes in patients.

## 2 MATERIALS AND METHODS

This study was performed at Al‐Zahra University Hospital, affiliated with Isfahan University of Medical Sciences and was approved by the research administration of Isfahan University of Medical Sciences and the Institutional Review Board. We reviewed hospitalized records of 285 patients admitted to the university hospital between March 2013 and August 2019 with diagnoses of cutaneous drug reactions. Following excluding patients with over 50% missing information, 195 patients were included in the study. The patients' demographics, clinical information, and pathologic data were extracted from medical records. Those parameters included age, gender, past medical history, medication history, DRESS, SJS, and TEN diagnoses. A clinical diagnosis was made by dermatologist. In addition, treatment regimens (steroids or intravenous immunoglobulin [IVIG]) and outcome information, including intensive care requirements, severe medical complications, or death, were obtained from medical records.

Statistical analysis was performed using Stata Software. For descriptive analysis, data are demonstrated as mean ± standard deviation or percentage (total number). To compare data, independent sample *T* test and Mann–Whitney test were used for continuous variables. For categorical data, *χ*2 was used. *p* Values less than 0.05 were considered significant.

## 3 RESULTS

Demographics, baseline clinical information, and clinical outcomes of the 195 included patients have been demonstrated in Table 1. The mean age of the patients was 39.5 (SD: 18.6; range: 1–88) years and 61% were female. Also, the median number of ACDR‐causing medications per patient is 2, and the most common medications are carbamazepine, lamotrigine, sodium valproate, and phenytoin. Among patients, 64% had skin biopsy. The rate of complications was 45% with DRESS, SJS, and TEN diagnosed in 26%, 47%, and 19%, respectively. The majority of patients (81%) were treated with oral steroids, while only 19% were treated with IVIG. In terms of outcome, 15% of studied patients received intensive care, and 5% died.

**Table 1:** Demographic and clinical characteristics of patients with skin reaction in a tertiary referral center in Isfahan, Iran.

Variable	Total; *N* = 195
Gender; *n* (%)	
Male	76 (39%)
Female	119 (61%)
Age (years); mean ± SD (range)	40 ± 19 (1–88)
Number of medication causing SJ; median [Q1–Q3]	2 [1–2]
Prescribed medication	
Carbamazepine	41 (21%)
Lamotrigine	33 (17%)
Sodium valproate	28 (14%)
Phenytoin	28 (14%)
Cefixime	15 (8%)
Penicillin	13 (7%)
Ciprofloxacin	13 (7%)
Metronidazole	12 (6%)
Gabapentin	11 (5%)
Biopsy; *n* (%)	124 (64%)
Confirmed cases; *n* (%)	22 (11%)
Complications; *n* (%)	87 (45%)
DRESS; *n* (%)	51 (26%)
SJS; *n* (%)	19 (47%)
TEN; *n* (%)	36 (19%)
Treated with steroid; *n* (%)	158 (81%)
Treated with IVIG; *n* (%)	37 (19%)
Received intensive care unit care; *n* (%)	30 (15%)
Mortality; *n* (%)	9 (5%)

Abbreviations: DRESS, drug reaction with eosinophilia and systemic symptoms; IVIG, intravenous immunoglobulin; SJS, Steven‐Johnson syndrome; TEN, toxic epidermal necrosis.

### 3.1 Impact of gender on clinical and outcome profile of patients

Baseline clinical information and outcome characteristics have been demonstrated based on gender in Table 2. Females were significantly older than males (41.6 ± 18.9 vs. 36.3 ± 17.7; *p* = 0.04). Males have higher rate of SJS (57.9% vs. 39.5%; *p* = 0.01; odds ratio [OR]: 2.10; 95% confidence interval [CI]: 1.17–3.78). There were significantly more males treated with IVIG (26.2% vs. 14.3%; *p* = 0.04; OR: 2.14; 95% CI: 1.04–4.42); however, clinical outcomes, such as rate of complication, intensive care requirement, or mortality, did not defer between genders.

**Table 2:** Clinical profile and outcomes of patients demonstrated by gender.

Variable	Male	Female	*p* Value*
Gender; *n* (%)	76 (39%)	119 (61%)	‐
Age (years); mean ± SD (range)	36.3 ± 17.7 (2−79)	41.6 ± 18.9 (1–88)	**0.04**
Number of medication causing SJ; median [Q1– Q3]	1.5 (1–2)	2 (1–3)	0.16
Prescribed medication	Carbamazepine (16), sodium valproate (11), phenytoin (14)	Carbamazepine (25), lamotrigine (23), sodium valproate (17), phenytoin (14), cefixime (11), ciprofloxacin (10)	n/a
Biopsy; *n* (%)	44 (57.9%)	80 (67.8%)	0.16
Confirmed cases; *n* (%)	8 (10.5%)	14 (11.9%)	0.77
Complications; *n* (%)	36 (47.4%)	51 (43.2%)	0.57
DRESS; *n* (%)	17 (28.6%)	34 (22.4%)	0.34
SJS; *n* (%)	44 (57.9%)	47 (39.5%)	**0.01**
TEN; *n* (%)	16 (21.1%)	20 (16.8%)	0.49
Treated with steroid; *n* (%)	62 (82%)	96 (81%)	0.90
Treated with IVIG; *n* (%)	20 (26.2%)	17 (14.3%)	**0.04**
Received intensive care unit care; *n* (%)	14 (18.4%)	16 (13.5%)	0.35
Mortality; *n* (%)	3 (4.0%)	6 (5.0%)	0.72

Abbreviations: DRESS, drug reaction with eosinophilia and systemic symptoms; IVIG, intravenous immunoglobulin; SJS, Steven‐Johnson syndrome; TEN, toxic epidermal necrosis.

* *p* Value resulted from Mann–Whitney/independent sample *t* test or chi‐square/Fisher's exact test analysis.

### 3.2 Impact of age on clinical and outcome profile of patients

Baseline clinical information and outcome characteristics have been demonstrated based on patients' age in Table 3. Age was a significant predictor of patient mortality with death rate ranging from 2% in the lowest quantile to 11% in the highest quantile of age. Patients at young age were also more likely to receive IVIG treatment, but there was no significant difference between age and other clinical or outcome information of patients.

**Table 3:** Clinical profile and outcomes of patients demonstrated by age.

Variables	First quantile (2–26 years)	Second quantile (27–35 years)	Third quantile (36–53 years)	Forth quantile (54–86 years)	*p* Value
Total	51	48	50	46	‐
Number of meds causing SJ, median (IQR)	0 (0–1)	0.5 (0–1)	0 (0–1)	0 (0–1)	0.08
Most common meds prescribed (number of patients taking)	Carbamazepine (12)	Sodium valproate (10), lamotrigine (10)	Carbamazepine (16)	Lamotrigine (7)	n/a
Experienced complications, % (*n*)	42% (21)	50% (24)	42% (21)	46% (21)	0.55
DRESS, % (*n*)	18% (9)	27% (13)	34% (17)	26% (12)	0.50
SJS, % (*n*)	65% (33)	40% (19)	40% (20)	41% (19)	0.10
TEN, % (*n*)	20% (10)	21% (10)	20% (10)	13% (6)	0.39
Treated with steroid, % (*n*)	78%	92%	78%	76%	0.73
Treated with IVIG, % (*n*)	26% (13)	17% (8)	24% (12)	9% (4)	0.02
Received ICU care, % (*n*)	18% (9)	13% (6)	16% (8)	15% (7)	0.97
Died, % (*n*)	2% (1)	0% (0)	4% (2)	11% (5)	**0.001**

Abbreviations: DRESS, drug reaction with eosinophilia and systemic symptoms; IVIG, intravenous immunoglobulin; SJS, Steven‐Johnson syndrome; TEN, toxic epidermal necrosis.

### 3.3 Impact of medications and past medical history on clinical and outcome profile of patients

Baseline clinical information and outcome characteristics have been tabulated in Table 4 based on the most common medications that the patients have been taking, which were known to cause ACDR. There was a markedly decreased rate of DRESS in patients taking metronidazole (8%) compared with other medications (18%−39%). On the other hand, metronidazole was associated with higher rate of SJS (75% compared with 39%–53%). Sodium valproate had the lowest association with TEN (7% vs. 15%–24%). There was also a notable increase in mortality in patients taking ciprofloxacin (15% vs. 0%–8%).

**Table 4:** Clinical outcomes of patients demonstrated by the prescribed medication and past medical conditions.

	Total number	DRESS (%)	SJS (%)	TEN (%)	Treated with prednisone (%)	Treated with IVIG (%)	Experienced complications (%)	Received ICU care (%)	Died (%)
Medication									
Carbamazepine	41	34	49	24	78	15	51	20	5
Cefixime	15	33	53	27	67	20	53	20	0
Ciprofloxacin	13	31	39	23	92	15	62	23	15
Gabapentin	11	18	46	27	82	27	46	9	0
Lamotrigine	33	24	42	18	88	18	42	9	6
Metronidazole	12	8	75	17	92	25	58	25	8
Penicillin	13	39	46	15	77	8	23	8	0
Phenytoin	28	33	41	19	85	15	37	7	0
Sodium valproate	28	33	44	7	74	7	48	7	0
Past medical condition									
Psychiatric Disease	41	32	49	12	93	17	46	12	2
Hypertension	32	19	44	22	66	13	53	19	3
Seizure	28	19	67	22	74	22	41	7	0
Diabetes	17	29	35	6	65	12	35	12	0
Neurological disorder (non‐seizure)	14	36	29	0	100	0	64	0	0
Autoimmune disease	12	50	8	8	92	8	50	0	0
Heart disease	12	17	50	33	75	8	75	33	25

Abbreviations: DRESS, drug reaction with eosinophilia and systemic symptoms; IVIG, intravenous immunoglobulin; SJS, Steven‐Johnson syndrome; TEN, toxic epidermal necrosis.

There is a notable decreased rate of SJS in patients with autoimmune disease compared with other conditions (8% vs. 29%–67%). The rate of TEN was lower for patients with diabetes (6%), non‐seizure neurological ailments (0%), or autoimmune diseases (8%). There was marked increased mortality in patients with heart disease (25% vs. 0%–3%).

### 3.4 Clinical and outcome profile of patients based on diagnosis of DRESS, SJS, and TEN

Clinical and outcome profiles of patients based on diagnosis of DRESS, SJS, and TEN have been demonstrated in Table 5. DRESS was noted in 26% of patients. There were significantly less ophthalmologic complications (0% vs. 16%; *p* = 0.002; OR: 0.84; 95% CI: 0.78–0.90). Patients with DRESS were significantly less likely to receive IVIG treatment (2% vs. 24%; *p* = 0.001; OR: 0.13; 95% CI: 0.03–0.55) or intensive care (2% vs. 19%; *p* = 0.008; OR: 0.17; 95% CI: 0.04–0.74). There was a trend towards less mortality in patients with DRESS (0% vs. 6%; *p* = 0.058).

**Table 5:** Clinical profile and outcomes of patients tabulated based on presence of severe complications, DRESS, SJS, or TEN.

Variables	DRESS			SJS			TEN		
	Positive	Negative	*p* Value	Positive	Negative	*p* Value	Positive	Negative	*p* Value
Total patients, *n* (%)	26% (50)	74% (145)	‐	47% (91)	53% (104)	‐	18% (35)	82% (160)	‐
Gender Male, % (n)	34%	41%	0.34	48%	31%	**0.01**	46%	38%	0.49
Age, mean ± SD	42 ± 19	39 ± 18	0.33	37 ± 20	42 ± 17	**0.03**	38 ± 18	40 ± 19	0.53
Ophthalmologic complications	0%	16%	**0.002**	22%	3%	**<0.001**	20%	10%	0.24
Treated with steroid, % (*n*)	88%	79%	0.13	87%	76%	0.05	77%	82%	0.73
Treated with IVIG, % (*n*)	2%	24%	**0.001**	23%	14%	0.08	69%	8%	**<0.001**
Received ICU care, % (*n*)	2%	19%	**0.01**	15%	14%	0.69	54%	6%	**<0.001**
Died, % (*n*)	0%	6%	0.06	6%	3%	0.36	14%	2%	**<0.001**

Abbreviations: DRESS, drug reaction with eosinophilia and systemic symptoms; IVIG, intravenous immunoglobulin; SJS, Steven‐Johnson syndrome; TEN, toxic epidermal necrosis.

Among studied patients SJS was diagnosed in 47%. Patients with SJS were more likely to be males (48% vs. 31%; *p* = 0.01) and were younger (37 ± 20 vs. 42 ± 17; *p* = 0.03). Ophthalmologic complications were significantly higher among SJS patients (87% vs. 2.9%; *p* < 0.001; OR: 9.48; 95% CI: 2.72–33.13). There was no significant difference in treatment or outcome between patients with or without SJS.

TEN was diagnosed in 18% of patients. Patients with TEN had significantly poorer outcomes, in which there were significantly higher rates of treatment with IVIG (69% vs. 8%; *p* < 0.001; OR: 24.67; 95% CI: 9.92–61.39), ICU care (54% vs. 6%; *p* < 0.001; OR: 16.09; 95% CI: 6.51–39.72), and mortality (14% vs. 2%; *p* < 0.001).

**Figure 1:**
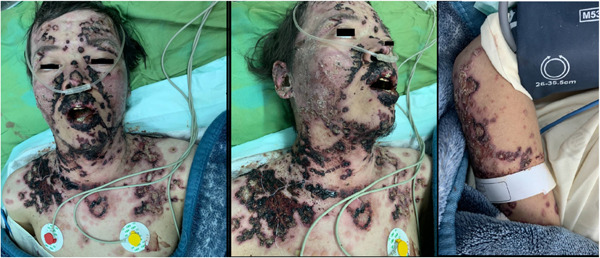
A case of overlapping toxic epidermal necrosis and Steven‐Johnson syndrome in a middle‐aged male with lupus.

**Figure 2:**
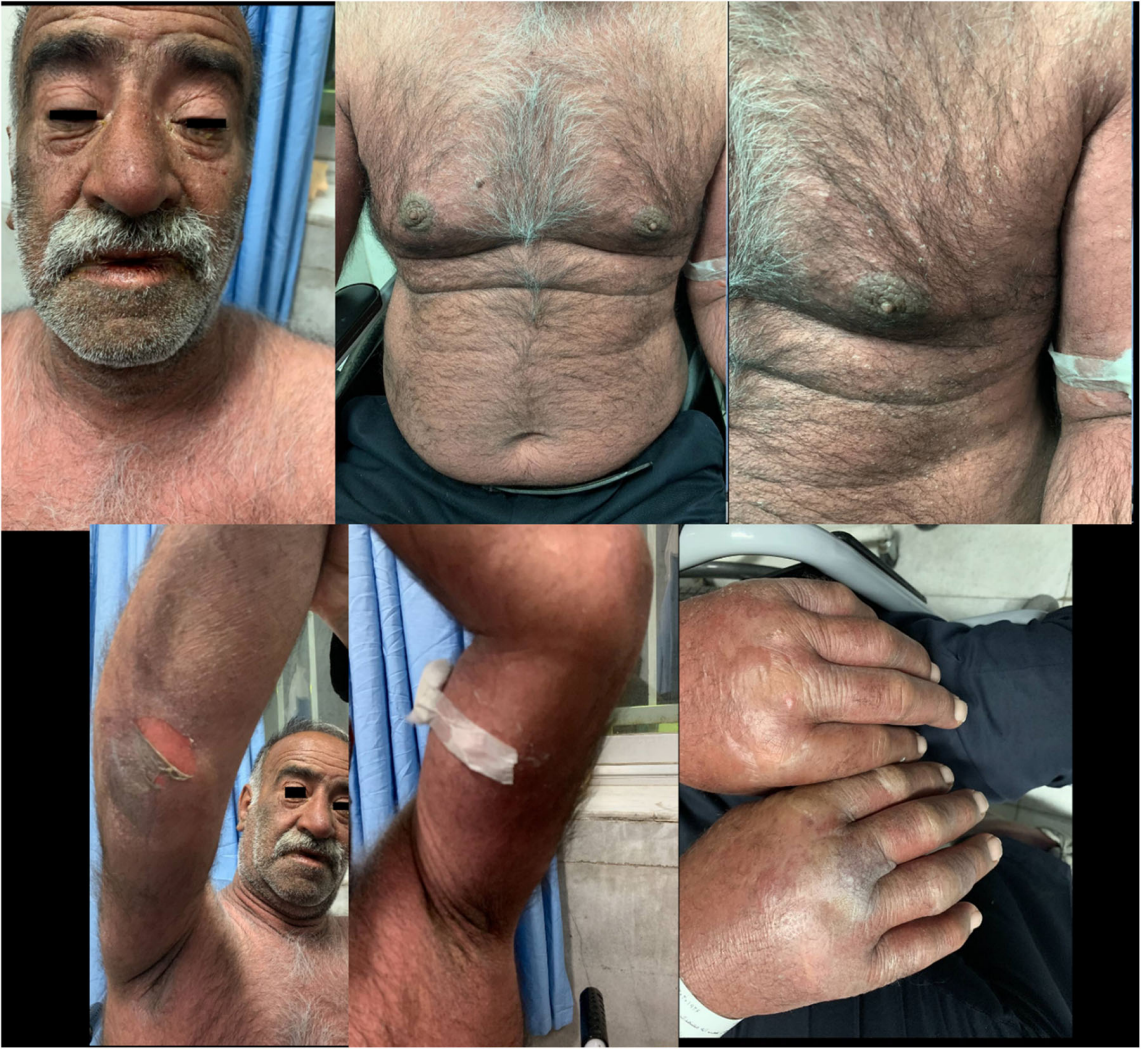
A case epidermal detachment with above 30% involvement, along with mucosal lesions.

**Figure 3:**
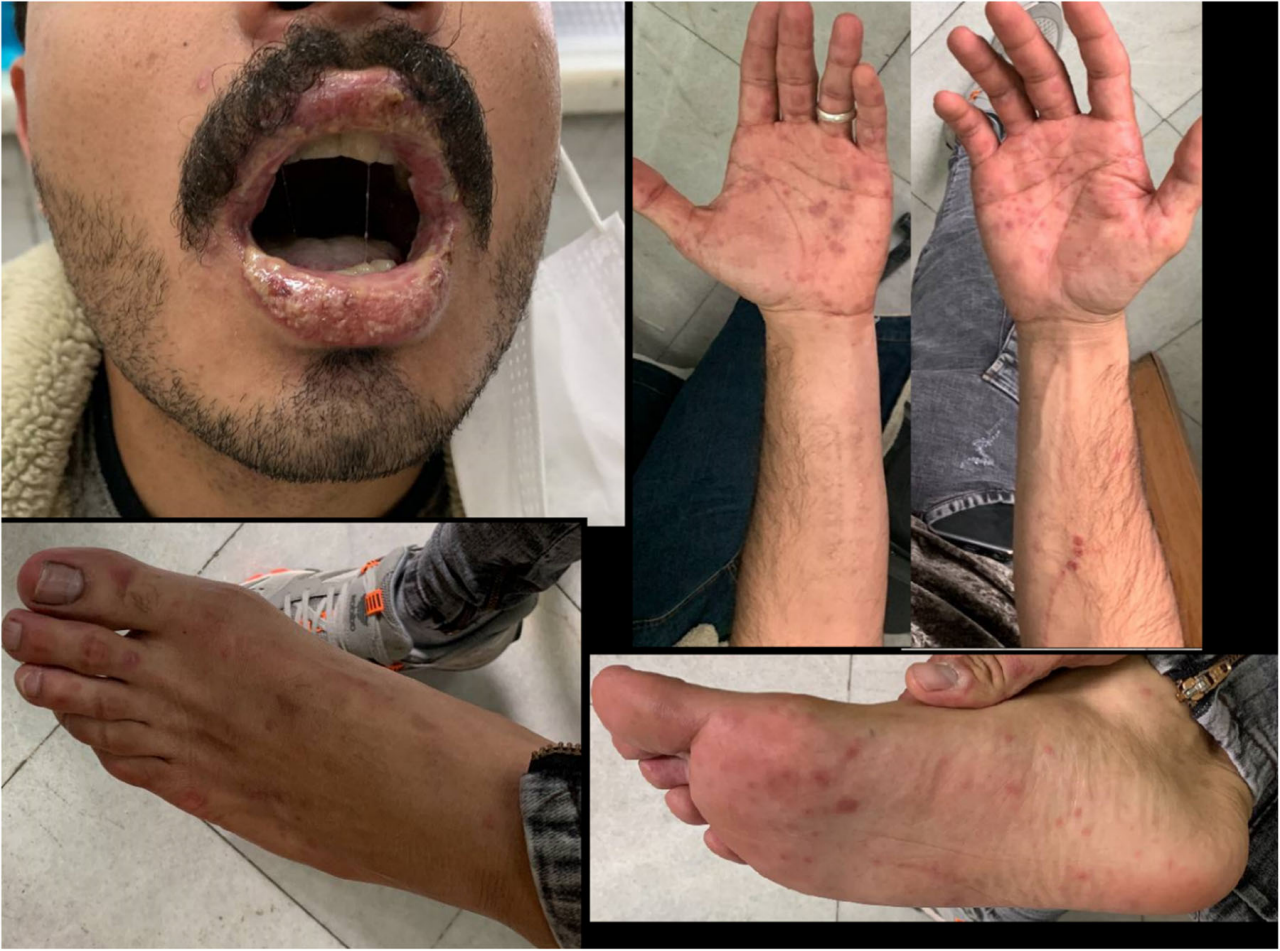
A case of Steven‐Johnson syndrome with mucous wounds, atypical target lesions, and epidermal detachment below 10% of the body.

We present clinical figures of patients in our study in Figures 1, 2, 3. Figure 1 presents a case of overlapping TEN and SJS in a middle‐aged male with lupus. Figure 2 demonstrates a case of epidermal detachment (>30%) along with mucosal lesions. Figure 3 illustrates a case of SJS with mucous wounds, atypical target lesions, and epidermal detachment below 10% of the body.

## 4 DISCUSSION

Adverse cutaneous reactions present in a spectrum including less severe immediate or delayed hypersensitivity reactions such as maculopapular exanthema, urticaria, and fixed drug reaction, to more rare but life‐threatening reactions such as TEN, SJS DRESS.^18^ In this study, we reported epidemiological, clinical and outcome information for the more severe ACDR, including SJS, TEN, and DRESS. We observed that there are more females included in this cohort study, which is compatible with prior studies showing more prevalence of ACDRs in the female population.^16^ This has been attributed to higher consumption of medications and more elderly patients, both precipitating factors for ACDRs, in the female population. Since our study is not population‐based and only involves patients admitted to a tertiary care center, we cannot comment on the community prevalence of severe ACDRs, but we can confirm that in Iran, ACDRs are more prevalent among females. In our cohort, female patients are significantly older than males, confirming higher age in females as a potential explanation for gender discrepancies. Males were more likely to receive IVIG, possibly explained by higher prevalence of SJS in male patients. There was no outcome difference between male and female patients.

The average age of patients with ACDRs is 40 years, which is younger than reported in European countries,^19^ and more similar to studies on the Asian population.^20^ This is most likely related to epidemiological and genetic similarities, but more studies are warranted to address age profile of patients with ACDRs. Age was highly associated with mortality (*p* < 0.001) with 6.5 times more mortality in the highest quantile compared with the lowest quantile. There was lower rate of treatment with IVIG in elder people, which could be potentially explained by concerns over the side effect profile of IVIG for elderly.

The immune system changes with age from a mainly Th2 pathway to a Th1 pathway, which has led to the development of specific management guidelines in elderly patients.^21^ An examination of cytokines in the peripheral blood of patients with IgE‐mediated allergic atopic dermatitis, conducted by Bozek et al., demonstrated a Th2 cytokine profile, predominantly featuring interleukin (IL)‐4, IL‐5, and IL‐13 in both young and elderly individuals. In contrast, elderly patients with low total IgE levels typically display a Th1 cytokine profile, characterized by elevated levels of IL‐17.^22^

We have antiepileptic medications to be the most common drug associated with ACDRs. Studies have shown while antibiotics and NSAIDS are the most commonly reported medications for all types of ACDRs,^14^, ^19^, ^23^ when limiting analysis or more severe ACDRs such as SJS or TEN, antiepileptic drugs such as carbamazepine, phenytoin, and lamotrigine are most frequently associated.^24^ In our cohort, rates of SJS, TEN, and SJS are 47%, 19%, and 26%, respectively. Our study notably reported higher rate of SJS for patients taking metronidazole, and lowest DRESS diagnosis for those patients. Ciprofloxacin was associated with the highest mortality rate among studied patients with ACDRs. The presence of underlying heart problems is another strong predictor of mortality in studied patients, but our sample size limited us in doing multifactor analysis to explain the higher rate of mortality in patients taking ciprofloxacin.

Patients with SJS were younger and more likely to be males, and they were more likely to have eye complications. On the other hand, patients with the diagnosis of TEN were more likely to receive IVIG and intensive care, and had a higher mortality rate.

The majority of patients (81%) have been treated with oral steroids, while 19% were treated with IVIG. In our studied cohort, 15% of patients required intensive care, and a 5% mortality rate was reported. Besides heart disease being associated with higher mortality, there was an intriguing association between the diagnosis of autoimmune diseases and lower rates of SJS and TEN. Given the immune‐mediated nature of SJS and TEN,^20^ immunomodulatory medications for patients with the autoimmune disease may explain lower rate of TEN or SJS. A higher sample size is required to enable the multi‐factorial analysis.

As mentioned, the limitations of our study included the small sample size and single‐center nature of our study, along with the retrospective design. Another limitation was the lack of laboratory evaluation, including C3, C4 C1INH, while also the documentation of SCORETEN among our patients. However, our results show the significance of ACDRs in our country while also providing valuable information for future multicentral and meta‐analytic studies.

## 5 CONCLUSION

We have shown that the demographic patterns of patients with ACDRs are similar to other reports from Asian countries. Mortality was around 5% and it was associated with diagnosis of TEN, older age, and baseline heart disease. In this report, we have evaluated demographic, clinical patterns, and outcome information in patients admitted to a referral center for ACDRs. This will help in understanding the clinical patterns of patients with ACDRs in Iran and help in predicting rates of complications, treatments, and outcomes in patients.

## AUTHOR CONTRIBUTIONS

**Mohammad Amin Moshayedi**: Resources; writing—original draft. **Ali Asilian**: Supervision. **Fatemeh Mokhtari**: Conceptualization; data curation; supervision.

## CONFLICT OF INTEREST STATEMENT

The authors declare no conflict of interest.

## ETHICS STATEMENT

The present study was approved by the Medical Ethics Committee of the Isfahan University of Medical Sciences. Based on the retrospective nature of the study, written informed consent was waived by the Ethics committee of the Isfahan University of Medical Sciences. Permission to carry out the study and access patient records was sought from the Isfahan University of Medical Science administrators, and the study was conducted in compliance in accordance with the relevant guidelines and regulations and the Declaration of Helsinki and was also approved by the ethics committee of the university. Written informed consent was obtained from the patients regarding the publication of their images and reports.

## TRANSPARENCY STATEMENT

The lead author Fatemeh Mokhtari affirms that this manuscript is an honest, accurate, and transparent account of the study being reported; that no important aspects of the study have been omitted; and that any discrepancies from the study as planned (and, if relevant, registered) have been explained.

## Data Availability

The data sets used and/or analyzed during the current study are available from the corresponding author upon reasonable request and with permission of the Research Ethics Committee of Isfahan University of Medical Sciences.
